# Supplementary material for: Retweet communities reveal the main sources of hate speech
Source: PLoS One. 2022 Mar 17;17(3):e0265602. doi: 10.1371/journal.pone.0265602 (PMC8929563; doi:10.1371/journal.pone.0265602)
Supplement: Attachment — Submitted filename: rebuttal.docx [file pone.0265602.s001.docx]

Dear Editor,

We are submitting a revised manuscript titled “Retweet communities reveal the main sources of hate speech”. We considered all the points raised by the two reviewers. We really appreciate very detailed and constructive comments provided by both reviewers and would like to thank them. Please find below the point-to-point answers to the reviewers’ comments.

Sincerely yours,

Igor Mozetic

**Reviewer #1:** **Data availability:** the authors, in line 372, claim that the dataset is available at the following link: https://www.clarin.si/repository/xmlui/handle/11356/1423. However, it looks like the repository is not publicly available, even if logged in through an institution. From a Google search about Slovenian Twitter dataset, I find the following dataset which seems employed in the study. The dataset I found is available at this link: https://www.clarin.si/repository/xmlui/handle/11356/1398

**Answer:**  The repository is now publicly available at the stated URL: <https://hdl.handle.net/11356/1423>.

**Reviewer #1:** **Reproducibility:** the reproducibility of the paper is not generally guaranteed, mainly for the absence of a public access to the dataset. In terms of the explanation of the methodology, the paper requires minor corrections to this part.

**Answer:**  The dataset is now publicly available. We also made publicly available the hate speech classification model for Slovenia tweets, at <https://huggingface.co/IMSyPP/hate_speech_slo>.

**Reviewer #1:** 1. Introduction - it seems to lack some brief introduction about the hate speech context, such as the problem it arises, the consequences, and similar. I would spend some lines at the beginning to briefly give some context to this topic to the reader.

**Answer:**  We added a brief introduction to the hate speech problems.

**Reviewer #1:** 2. line 2 and 3: I find the first clause a strong statement, especially if we consider where it is placed and the lack of literature in support. I encourage you to rephrase it and/or find works that can sustain this opening.

**Answer:**  We added references to the most relevant competitions and approaches to automated hate speech detection.

**Reviewer #1:** 3. Twitter data, line 111 and 112: "various social phenomena" could be less vague by citing some influential works that provide an example of the aforementioned phenomena. I suggest to cite works like this one: https://journals.plos.org/plosone/article?id=10.1371/journal.pone.0234689

**Answer:**  We added references to some influential works in addition to the proposed citation.

**Reviewer #1:** 4. Results and discussion, Table 1: "Dec. 2017" is ambiguous. In the data collection section, it is mentioned that TweetCat started to collect Slovenian tweets at the end of 2017 but, at the same time, the Twitter data covered the period from 2018-2020. Therefore, would spend some words to clarify the presence of this "Dec. 2017" term.

**Answer:**  The TweetCat tool started collecting Slovenian Twitter data on August 28, 2017. We sampled the training data for learning classification models from December 1, 2017 until February 2020. And for the networks analysis and model application we choose clear 3-year period, from January 1, 2018, until December 28, 2020. We updated the Data collection paragraph.

**Reviewer #1:** 5. line 131 and 132: "Out of [...] not". I suggest rephrasing it to be less qualitative.

**Answer:**  We added approximate quantities in the text, detailed numbers are in Tables 1 and 2.

**Reviewer #1:** 6. Conclusions, line 325: the part about hate speech detection should have been placed before the community one in order to maintain the same order with the Results section

**Answer:**  We changed the order of paragraphs, as suggested.

**Reviewer #1:** 7. Methods: in Cohen's h, I would move the term "(probabilities)" to the first occurrence of proportions in the caption.

**Answer:**  Changed.

**Reviewer #1:** 8. line 382: "Cohen provides [..] interpretation", I think it would be more sound to cite again the paper providing this rule of thumb.

**Answer:**  We added the reference, including the page numbers with the rule-of-thumb.

**Reviewer #1:** Twitter data, line 115: "levels of hate speech" refers to a result of the ML part. At this point, it should be pointed out how those levels were measured.

**Answer:**  We replaced levels by types, and added a clarification that hate speech types were determined automatically by our classification model.

**Reviewer #1:** Communities and hate speech, line 163: "tightly linked" is too vague. Which measures or analysis led to the definition of "tightly linked"? A better explanation is required, especially in this part where the creation of super-communities is crucial for the results presented below in the paper.

**Answer:**  We added a paragraph and an informal definition of super-communities. A formal definition and design of a multi-stage super-community detection algorithm is, to the best of our knowledge, an open research problem.

**Reviewer #1:** Twitter users and hate speech, line 250: the clause "the results are consistent with Figure 3" requires to be rephrased in order to provide more evidence about this consistency.

**Answer:**  We rephrased the sentence and added actual fractions of unacceptable tweets posted by the three communities.

**Reviewer #1:** Line 267: "A prevailing community [...] is also determined". How?

**Answer:**  We rephrased the sentence to clarify that the overall community membership is determined from communities at individual timepoints, when the user did not switch between the communities.

**Reviewer #1:** Hate speech classification, line 386-387: "Supervised machine learning requires a large set of examples labeled for hate speech" is ambiguous. Probably, you are only referring to the context of hate speech. However, this sentence should be motivated by citing works that sustain this "rule of thumb".

**Answer:**  We rephrased the sentence and added a reference.

**Reviewer #1:** Methods - The heuristic algorithm proposed to find the k timepoints requires better explanation and editing. It could be provided in a pseudocode way and/or by providing a visualization of a general iteration

**Answer:**  We clarified the description of the heuristic algorithm and added a simple visualization of the general iteration step.

**Reviewer #1:** Figure 1: axis labels are required.

**Answer:**  Axis labels were added.

**Reviewer #1:** Figure 3: y-axis label and legend title are required.

**Answer:**  The label and the legend title were added.

**Reviewer #1:** Figure 4: y-axis and legend title are required. Furthermore, its caption should be modified in order to be less colloquial and more informative.

**Answer:**  The label and the legend title were added, and we expanded the caption.

**Reviewer #1:** Figure 5: I am not convinced by this plot. It is too qualitative and compares different metrics. Cohen's h gives an answer on this side, but it requires checking the table containing the results, meaning that the figure itself does not provide precise information. It is visually hard to understand the differences between communities on the metrics proposed if a scale is missing. Therefore, the explanation proposed from lines 212 to 231 may be not so clear to the reader as the paper states. My proposal is to switch to a figure containing a bar plot for each metric of the different measurement units. In the end, a title for the legend is missing.

**Answer:**  We replaced the figure with bar plots, added the legend title, and expanded the caption.

**Reviewer #1:** Figure 6: I would add the explanation of the vertical line in the caption.

**Answer:**  The caption was expanded to include an explanation of the vertical bars.

**Reviewer #1:** Figure 7: The figure lacks a legend and an x-axis label. The caption should be more descriptive, briefly describing the results and being less colloquial with the definition of the "refined subtypes".

**Answer:** We added the legend, but did not add the x-axis label since the x-axis is categorical. We also expanded the caption to be more informative.

**Reviewer #1:** 1. Abstract: the usage of "however" in the last sentence is ambiguous. The sentence in which this word is used does not present any contradiction with respect to the previous one but, in some way, it enforces the statement. Therefore, I would suggest changing to "Indeed", "In fact" or similar.

**Answer:**  We replaced “However” with “In fact”.

**Reviewer #1:** 2. Related work, line 29: change to Related works.

**Answer:**  Changed.

**Reviewer #1:** 3. line 33: I find the term "Work" colloquial in the context of a paper. I suggest changing to "Results" since we rely on the outcomes of the works.

**Answer:**  Changed.

**Reviewer #1:** 4. line 44: since only one further work is cited, I would suggest modifying "Another" to "An additional", becoming less colloquial at the same time.

**Answer:**  Modified.

**Reviewer #1:** 5. Results and discussion, line 104: "The combination then" should be changed to "This combination"

**Answer:**  Changed.

**Reviewer #1:** 6. Table 1: I would replace the term "Same" with the periods, despite their redundancy.

**Answer:**  Replaced.

**Reviewer #1:** 7. Line 145. I would change "we use 24 week retweet windows" to "we use a time span of 24 weeks for our retweet networks"

**Answer:**  Changed.

**Reviewer #1:** 8. Communities and hate speech, line 155: "elsewhere" is too colloquial. It should be more descriptive, briefly describing the context in which the methods described were used. Furthermore, given the importance of the claim, I suggest to cite a peer-reviewed work instead of an Arxiv one. My proposal is the following: **[missing]**

**Answer:**  We added a brief description of our previous work with updated reference. The paper cited is already accepted to PLoS ONE, but not yet published. We changed the citation, but kept the arXiv reference (where a link to the journal paper will be published when available).

**Reviewer #1:** 9. line 171: change "," to ", namely" or similar.

**Answer:**  Changed.

**Reviewer #1:** 10. line 206: the comma should be replaced with a period since you are explaining two different standalone concepts.

**Answer:**  Replaced.

**Reviewer #1:** 11. line 225-227: sounds too mechanical to me. There is an overabundance of subordinates clauses.

**Answer:**  We rephrased the paragraph to make it clearer.

**Reviewer #1:** 12. Twitter users and hate speech, line 265: what does the term "Selected" refer to?

**Answer:**  We rephrased the sentence to clarify that we selected the top users according to their h-index influence ranks.

**Reviewer #1:** 13. Conclusions, line 310-311: "consistent with the findings elsewhere" too colloquial and general.

**Answer:**  We expanded the line to be more specific.

**Reviewer #1:** 14. line 312: I would change "difference" to "differences"

**Answer:**  Corrected.

**Reviewer #1:** 15. Hate speech classification, line 386: "elsewhere" is too colloquial. Please, give more context. Furthermore, the cited work lacks of a journal or conference name. Please provide more details about it.

**Answer:**  We removed this reference because the paper is still under review and is not available on arXiv.

=================================================================================

**Reviewer #2:** L 121. The authors often differentiate between unacceptable and acceptable tweets, but provide no examples of each tweet (also for subcategories of the unacceptable tweets). Could the author provide, if possible, examples in a supplementary file?

**Answer:**  We do not provide examples of hate speech in the paper because we consider this not to be the main focus of this paper. However, we made the hate speech classifier for Slovenian tweets openly available at: <https://huggingface.co/IMSyPP/hate_speech_slo> where any short text in Slovenian can be automatically classified in one of the four (sub)categories of hate speech. All the Slovenian Twitter data 2018-2020 are also made available.

**Reviewer #2:** I understand the authors want to summarize all methods and definition in one section after the main discussion, but, it may be helpful to have some (very short) explanation also in the result and discussion section. Sometimes they do that e.g. line 147, sometime they do not e.g. line 232 and line 241 (short explanation required).

**Answer:**  We added a brief explanation of Cohen’s h and retweet h-index to the main section.

**Reviewer #2:** L 265. The authors categorize the most important tweeters (890 users), but do the authors know the concentration amongst all users? In other words, could they also provide the users' concentration index, also independently from the classification of their tweets?

**Answer:**  We added a table with the Gini coefficients of h-index influence for the three largest communities. We also experimented with the Herfindahl-Hirschman index, but did not get any meaningful results (the values a too low for large numbers of users).

**Reviewer #2:** L 284 & L 316. Do the authors know how many profiles have been suspended by Twitter or closed over time?

**Answer:**  We added a footnote: On the global level, Twitter is suspending an increasing number of accounts, with 776,000 accounts suspended in the second half of 2018, and one million accounts suspended in the second half of 2020.

**Reviewer #2:** L 334. The authors mention the subcategories of unacceptable tweets, it is unclear here if they are able to catch all the categories of tweets or if they are able to catch, consistently with the benchmark of the human annotators, a coherent (and consistent) amount of unacceptable tweets, if so, it would be helpful to know (e.g. extend the table 7) the classification statistics for each subclass of unacceptable, maybe in an supplementary appendix.

**Answer:**  Each of the collected tweets is classified into one (sub)category. We removed F1 scores from Table 8 (former Table 7). We added Table 9 with F1 scores for the main categories (acceptable and unacceptable), and all the subcategories of hate speech. We also added Table 10 with the distribution of hate speech subcategories, separately for the original tweets and the retweets.

**Reviewer #2:** L 406. About the human annotator training, did authors share uniformly the dataset to the annotators(e.g., 5,000 tweets per annotator)?

**Answer:**  Yes, the tweets were uniformly distributed between the ten annotators. We amended the text accordingly.

**Reviewer #2:** L 417. I understand the use of the Krippendorff's alpha but, do the authors considered other measures - such as the Fleiss's k - to investigate the (dis)agreement between the raters?

**Answer:**  We did not use other (dis)agreement measures because alpha is claimed to be a generalization of several of them (Scott’s Pi, Fleiss’ K, Spearman’s rank correlation coefficient, and Pearson’s intraclass correlation coefficient). We amended the text accordingly.

**Reviewer #2:** L 458, could the authors give (beyond the reference cited) a brief definition of the approach (2 rows max), to clarify the methodology?

**Answer:**  We added a brief description of Bayesian significance testing as applied in machine learning to compare different models.

**Reviewer #2:** About citation #16 there is no reference available

**Answer:**  This paper is still under review, we removed the citation.
