# Supplementary material for: Retweet communities reveal the main sources of hate speech
Source: PLoS One. 2022 Mar 17;17(3):e0265602. doi: 10.1371/journal.pone.0265602 (PMC8929563; doi:10.1371/journal.pone.0265602)
Supplement: Attachment — Submitted filename: rebuttal-v4.docx [file pone.0265602.s002.docx]

Dear Editor,

We are submitting a revised manuscript titled “Retweet communities reveal the main sources of hate speech”. Please find below the point-to-point answers to the concerns raised.

Sincerely yours,

Igor Mozetic

1) Please provide further details in the Methods section on how hate speech, and the individual hate speech classes, are defined in this study.

**Answer:**  We expanded the section on Hate speech classification to include detailed criteria and definitions of hate speech classes. We added two references [27, 28] where the original annotation schema was proposed, and a reference to the IMSyPP project annotation guidelines [29].

2) Please clarify the criteria the annotators used to label the tweets into the different classes.

**Answer:**  The annotators were instructed to follow the annotation guidelines (ref [29]), and there was an initial trial period. The quality of annotations was monitored during the process by measuring the annotator self-agreement (when the same tweet was presented to an annotator twice at different times), and the inter-annotator agreement (when the same tweet was presented to two different annotators). The annotators also had previous experiences in annotating Facebook posts for the same hate speech classes as used in this study.

3) Please clarify how the communities were identified, and how the labels were chosen to describe the different communities identified.

**Answer:**  The communities are defined by their members and are detected by the community detection algorithm. The labels assigned to the communities are just shorthands to facilitate discussion. We considered who are their most influential members, what type of users are they (politicians, journalists, public figures, …), and what do they tweet about – on this basis we assigned the labels. This is described in the revised manuscript.
